# The spatial and temporal patterns of falciparum and vivax malaria in Perú: 1994–2006

**DOI:** 10.1186/1475-2875-8-142

**Published:** 2009-06-27

**Authors:** Gerardo Chowell, Cesar V Munayco, Ananias A Escalante, F Ellis McKenzie

**Affiliations:** 1 Mathematical, Computational & Modeling Sciences Center, School of Human Evolution and Social Change, Arizona State University, Tempe, Arizona, USA; 2 Division of Epidemiology and Population Studies, Fogarty International Center, National Institutes of Health, Bethesda, Maryland, USA; 3 Ministry of Health, Perú Jr Camilo Carrillo 402, Jesús María-Lima 11, Perú; 4 School of Life Sciences, Arizona State University, Tempe, Arizona, USA

## Abstract

**Background:**

Malaria is the direct cause of approximately one million deaths worldwide each year, though it is both preventable and curable. Increasing the understanding of the transmission dynamics of falciparum and vivax malaria and their relationship could suggest improvements for malaria control efforts. Here the weekly number of malaria cases due to *Plasmodium falciparum *(1994–2006) and *Plasmodium vivax *(1999–2006) in Perú at different spatial scales in conjunction with associated demographic, geographic and climatological data are analysed.

**Methods:**

Malaria periodicity patterns were analysed through wavelet spectral analysis, studied patterns of persistence as a function of community size and assessed spatial heterogeneity via the Lorenz curve and the summary Gini index.

**Results:**

Wavelet time series analyses identified annual cycles in the incidence of both malaria species as the dominant pattern. However, significant spatial heterogeneity was observed across jungle, mountain and coastal regions with slightly higher levels of spatial heterogeneity for *P. vivax *than *P. falciparum*. While the incidence of *P. falciparum *has been declining in recent years across geographic regions, *P. vivax *incidence has remained relatively steady in jungle and mountain regions with a slight decline in coastal regions. Factors that may be contributing to this decline are discussed. The time series of both malaria species were significantly synchronized in coastal (ρ = 0.9, P < 0.0001) and jungle regions (ρ = 0.76, P < 0.0001) but not in mountain regions. Community size was significantly associated with malaria persistence due to both species in jungle regions, but not in coastal and mountain regions.

**Conclusion:**

Overall, findings highlight the importance of highly refined spatial and temporal data on malaria incidence together with demographic and geographic information in improving the understanding of malaria persistence patterns associated with multiple malaria species in human populations, impact of interventions, detection of heterogeneity and generation of hypotheses.

## Background

Malaria is the most significant vector borne disease of humans; it is the direct cause of approximately one million deaths each year, though it is both preventable and curable. Most malaria in humans is due to *Plasmodium falciparum *and *Plasmodium vivax *[1], which are generally transmitted by the same species of *Anopheles *outside Africa.

Nowadays, developing strategies for malaria elimination is considered a global health priority [2]. Although reaching such ambitious goal may not be possible, the available tools will allow reducing the global burden of malaria if they are properly deployed. Thus, a key element for malaria elimination programmes is a good understanding of the malaria transmission dynamics in time and space. This is especially important in areas with low and intermediate seasonal transmission, such as those found in South America. There, previous elimination efforts, with the use of chloroquine and DDT, succeeded in vast areas during the 1970's. While most of the attention has been devoted to *P. falciparum *in Africa, an important element in malaria elimination programs outside Africa is *P. vivax*, a major challenge given that it requires an extended treatment in order to eradicate hypnozoites. Unfortunately, there are still a limited number of studies considering the joint dynamic of these two parasites, *P. falciparum *and *P. vivax*, in time and space. In this investigation, the temporal and spatial trends of these parasites in Perú are explored as an example of the complex dynamic of these parasites in areas with seasonal malaria outside Africa. In South America, severe malaria caused by *P. falciparum *formerly occurred only at Ecuadorian, Colombian, and Brazilian borders while *P. vivax *was the most important malaria parasite in the region in terms of its morbidity [3]. However, the incidence of falciparum malaria increased dramatically in Perú in the early 1990s, with a seven-fold increase of malaria incidence between 1990 and 1996 [4]. Nowadays, Perú is ranked second after Brazil in terms of the number of malaria cases in South America. Specifically, the Northern Peruvian Amazon (Loreto department comprising about one fourth of the total surface area of Perú), with a population clustered in town and villages throughout the Amazon tributary system, has been the epicenter of the malaria epidemic since the early 1990s [3]. While cases of falciparum malaria occur mostly in the jungle areas of Perú, *P. vivax *malaria is endemic in the coastal and mountain as well as jungle areas [5]. Moreover, *P. vivax *has replaced *P. falciparum *as the dominant species since 2000 [3].

In this paper, the weekly time series of malaria notifications from the Ministry of Health of Perú are used to analyse the spatial and temporal trends of *P. falciparum *(1994–2006) and *P. vivax *(1999–2006) malaria across jungle, mountain and coastal areas. The goal here is to increase the understanding on periodicity patterns, persistence and spatial heterogeneity associated with *P. falciparum *and *P. vivax *malaria at different spatial scales including national, geographic and province levels. Findings could shed light to public health authorities on how to effectively distribute resources for malaria control programmes at the national level.

## Methods

**Figure 1:**
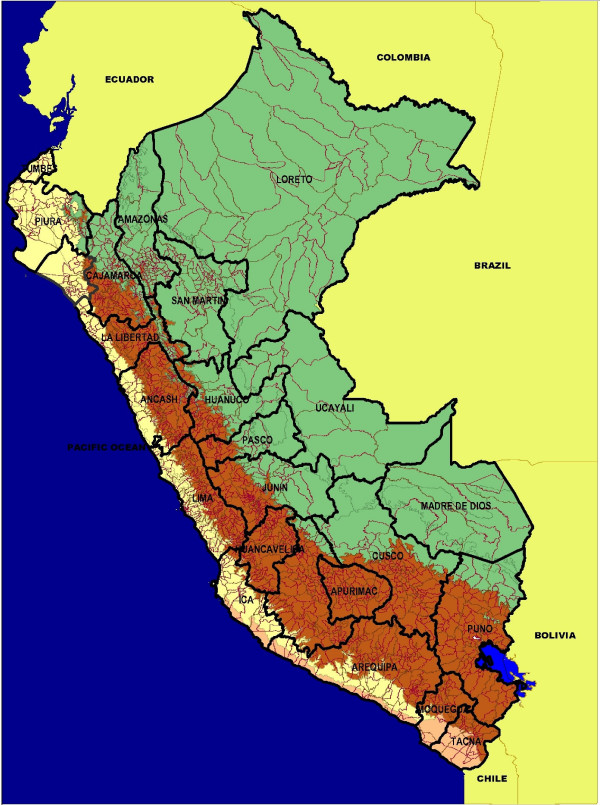
**Map of Perú with political boundaries of 195 provinces and 25 regions**. The geography of Perú covers a range of features, from a western coastal plain (yellow), the Andes Mountains in the center (brown), and the eastern jungle of the Amazon (green). The total population of Perú is about 29 million heterogeneously distributed in an area of 1,285,220 km^2^.

Perú is located on the Pacific coast of South America between the latitudes: -3 degrees S to -18 degrees S. It shares borders with Bolivia, Brazil, Chile, Colombia, and Ecuador (Figure 1). Perú total population is about 29 million, heterogeneously distributed over a surface area of 1,285,220 km^2 ^with distinctive landscapes including a western coastal plain, the eastern jungle of the Amazon and the Andes Mountains separating coastal and jungle areas (Figure 1). The country is divided into 25 administrative regions composed of 195 provinces [6].

Perú's weather varies from tropical by the Amazon to temperate and glacial in the Andes mountain range, while it is dry by its coast. Specifically, the jungle (rainforest) has two main seasons namely a May-October dry season, with high temperatures and warm nights (with the exception of June, when temperatures can drop significantly at night) and a November-April rainy season, with temperatures reaching 36°C and heavy rainfall that causes rivers to rise considerably leading to flooding on the smaller tributaries [3]. The mountain range region also has a May-October dry season, but characterized by clear, sunny days and cold nights, and a November-April rainy season, with heaviest rainfall during the months of January and February and mild daytime temperature that drops at night. The coastal region has an April-November winter season, with cloudy and cool days, and a hot and dry summer December-March, except for the northern coast with higher temperatures and rainfall in the summer.

### Data sources

The Directorate General of Epidemiology of Perú's Health Ministry is in charge of epidemiological surveillance, which is carried out from a network of over 6,000 geographically distributed notifying units. Perú's epidemiological passive surveillance system includes 95% of the health centers, and has collected weekly malaria data since 1994. All symptomatic individuals presenting fever, chills, headache and general malaise that had been in a malaria endemic area are routinely tested for the malaria parasites by microscopy on site or at the closest accredited laboratory. Notification of malaria cases is mandatory and is carried out weekly. Malaria patients receive free treatment in accordance with national guidelines. *Plasmodium falciparum *symptomatic cases have been reported since 1994 while mandatory notification of *P. vivax *did not start until 1999. For each of the provinces the weekly number of cases at reported symptom onset of *P. falciparum *during the period 1994–2006 and *P. vivax *cases during the period 1999–2006 were obtained from the Health Ministry's Directorate General of Epidemiology. A total of 163 provinces reported malaria cases sometime during the period of interest (1994–2006), of which only 78 reported *P. falciparum *cases. Reports of mixed infections were rare.

### Population, geographic, and climate data

The population size of the Peruvian provinces during the years 1994–2006 was obtained from the National Institute of Statistics and Informatics of Perú [7]. The population density of each province (people/km^2^) is estimated by dividing the province population size by the surface area (km^2^) [8]. These averages ranged from a mean of 22.3 people/km^2 ^in the mountain range, to 12.38 in the jungle areas, and 172 in the coastal areas (Additional file 1). Each province is classified according to its geographic location as coastal (n = 77), mountain (n = 89), or jungle area (n = 29, see Figure 1).

Weekly climate time series were obtained from meteorological stations located in 28 provinces distributed across Perú during the period 1994–2006. Out of the 28 meteorological stations, 13 were located in coastal areas, eight in mountain areas, and seven in jungle areas. Climate data included mean, minimum, and maximum temperature (Fahrenheit) and precipitation (inches) [9].

### Time series analysis of *P. falciparum *and *P. vivax *malaria across geographic regions

Periodic patterns were analysed through wavelet spectral analysis [10-12], disease persistence and critical community size [13-16], and spatial heterogeneity by applying two methods derived from econometrics and previously applied in infectious disease epidemiology, the Lorenz curve and the summary Gini index [see [17-20]].

#### Wavelet spectral analysis

Wavelet time series analysis [10-12] has received increasing attention in the last few years as a means of disentangling the non-stationary spatial and temporal dynamics of infectious disease and ecological systems [21-24]. In the temporal evolution of the number of disease cases, the presence of an annual cycle would indicate a single epidemic period per year in the time series while, for example, a biennial pattern characterizes an epidemic period every two years. Wavelet time series analyses are primarily powerful in detecting changes in epidemic periodicity (e.g., switch from biennial to annual cycles). Here the wavelet power spectrum (using the Morlet wavelet as in previous studies [21-24]) was used to investigate variations in the dominant periodic cycles across the time series using freely available software [25]. The weekly time series were log transformed to manage the variability in the amplitude of the time series.

#### Critical community size

Several studies have addressed the problem of disease persistence as a function of community size in island and non-island populations (e.g. [13-16,26]). It is therefore of interest to identify a "critical" community size, across geographic regions, above which malaria typically persists. Determining the effective or critical community size for a particular "invasion" is a complex matter because of variations in herd immunity, immigration rates, the possibility of disease reintroductions in the population, and the nature of human interactions. The persistence of malaria was assessed from the proportion of weeks with no malaria reports for each of the provinces in the weekly time series as has been used in previous studies [26].

The possibility of a critical population density (people per km^2^) was also evaluated, but no-significant association was found between population density and the proportion of weeks with no malaria reports (*P. falciparum *or *P. vivax*).

#### Spatial heterogeneity

Spatial variations in attack rates have not been extensively studied. Here, the Lorenz curve and associated summary Gini index at the province level, an approach derived from econometrics, are used to quantify spatial heterogeneity of malaria [17-20]. The Lorenz curve is a graphical representation of the cumulative distribution function of a probability distribution; in this case it represents the proportion of malaria cases associated with the bottom *y*% of the population comprised by the provinces previously ranked by case incidence rates. Equal attack rates (no heterogeneity) result in a first diagonal Lorenz curve. On the other hand, perfectly unbalanced distributions give rise to a vertical Lorenz line (maximum heterogeneity). Most empirical attack rate distributions lie somewhere in-between.

The Gini index summarizes the statistics of the Lorenz curve (ranging between 0 and 1). It is calculated as the area between the Lorenz curve and the diagonal representing no heterogeneity. A large Gini index indicates high heterogeneous attack rates, that is, a situation where the highest attack rates are concentrated in a small proportion of the population. A Gini index of zero indicates that attack rates are directly proportional to population size (no heterogeneity).

## Results

### Temporal patterns of *P. falciparum *and *P. vivax *at the national level

**Figure 2:**
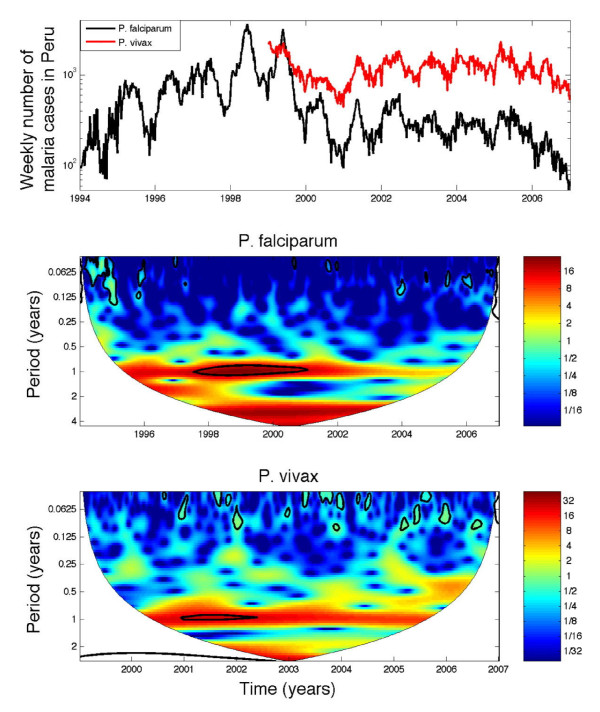
**The weekly time series of the number of *Plasmodium falciparum *(1994–2006) and *P. vivax (1999–2006) *in Perú during the period 1994–2006 (in logarithmic scale) and the corresponding wavelet power spectrum for both malaria time series**. For both time series, annual cycles have the highest power. There is also a strong triennial pattern for the *P. falciparum *series during 1998–2003 and a strong biennial cycle for *P. vivax *during 2002–2004.

Overall the mean annual incidence rates of *P. falciparum *in Perú during 1994–2006 ranged from 57.3 cases per 100,000 individuals in 2006 to 686 cases per 100,000 individuals in 1998 while the mean annual incidence of *P. vivax *during 1999–2006 ranged from 201 cases per 100,000 in 2000 to 461 cases per 100,000 in 1999. Figure 2 shows the overall temporal trend of the malaria burden due to *P. falciparum *(1994–2006) and *P. vivax *(1999–2006). For both species, wavelet time series analysis indicated that annual cycles have the highest power. Results also suggest a triennial pattern for the *P. falciparum *series during 1998–2003 and a strong biennial cycle for *P. vivax *during 2002–2004 (Figure 2). Inspection of the aggregated data at the national level (1999–2006) indicates that both malaria species follow a significantly synchronized dynamical process. In fact, the weekly counts aggregated at the national level of *P. vivax *and *P. falciparum *are significantly correlated (Spearman ρ = 0.62, P < 0.001). However, despite the high levels of apparent temporal synchronization in the time series of both malaria species, there are important differences in the magnitude and periodicity in the incidence of *P. falciparum *and *P. vivax *malaria when the time series are stratified into geographic regions and provinces as shown below.

### Temporal trends of *P. falciparum *and *P. vivax *malaria by geographic region

**Figure 3:**
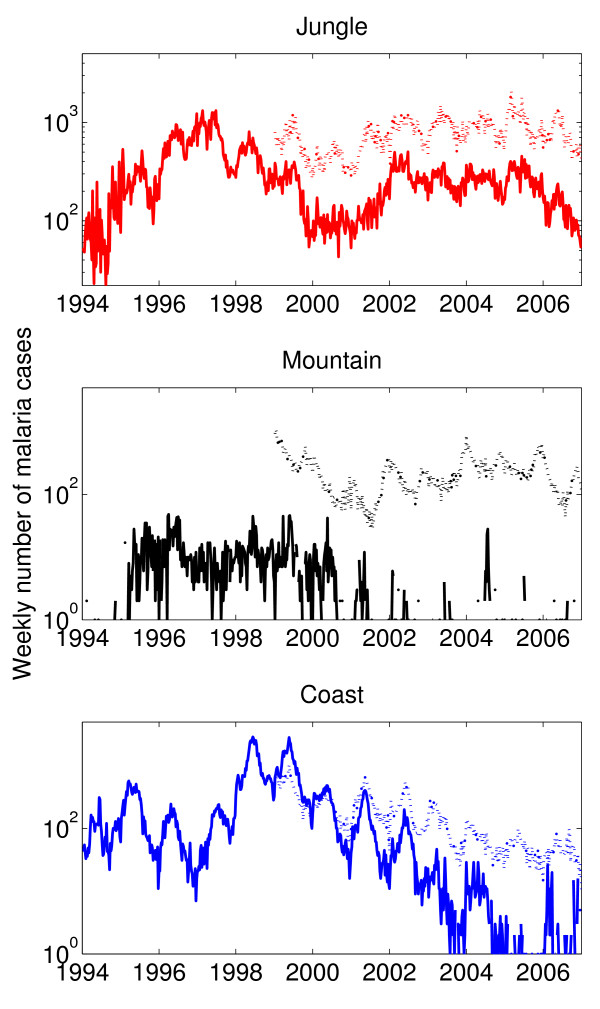
**The weekly number of cases due to *Plasmodium falciparum *(solid line) and *P. vivax *(dotted line) in Perú stratified by geographic region (jungle, mountain range, coast) during the period 1994–2006 (notification of *P. vivax *did not start until 1999)**. A decline in the number of malaria cases due *to P. falciparum *in the mountain and coastal areas can be observed in the last few years while *P. vivax *has only been declining in coastal regions in the last few years.

The weekly number of malaria cases due to *P. falciparum *and *P. vivax *malaria in jungle, mountain and coastal regions are displayed in Figure 3. The annual rates of *P. falciparum *have significantly declined across all geographic regions between 1999 (702.6, 10 and 268.5 cases per 100,000 people in jungle, mountain and coastal regions, respectively) and 2006 (213, 0.2 and 0.9 cases per 100,000 people in jungle, mountain and coastal regions, respectively). On the other hand, the annual rates of *P. vivax *have remained relatively steady in jungle and mountain regions during the last few years of the study period (1283.3 and 96.6 cases per 100,000 people in 2006, respectively) while declining in coastal regions. Overall, *P. vivax *has been the dominant malaria species affecting mountain regions since year 2000, with only brief and small peaks of *P. falciparum *occurrin *g *in the last few years (Figure 3).

### Periodicity and correlation of malaria time series across geographic regions

Between 1994 and 1998, the incidence of *P. falciparum *in the jungle region was moderately correlated with that in the mountain range region (ρ = 0.52, P < 0.0001) but not in the coastal region (ρ = 0.07, P = 0.28). Similarly, the incidence of *P. falciparum *in mountain range areas was only weakly positively correlated with coastal areas (ρ = 0.24, P < 0.0001) during the same period. During 1999–2006, when *P. falciparum *incidence started to decline, the incidence of *P. falciparum *in the mountain regions was correlated with that in the coastal areas (ρ = 0.62, P < 0.0001). In contrast, in the same period the dynamics of *P. falciparum *were weakly negatively correlated in jungle and coastal regions (ρ = -0.13, P = 0.006) and uncorrelated between jungle and mountain regions (ρ = 0.008 P = 0.87).

**Figure 4:**
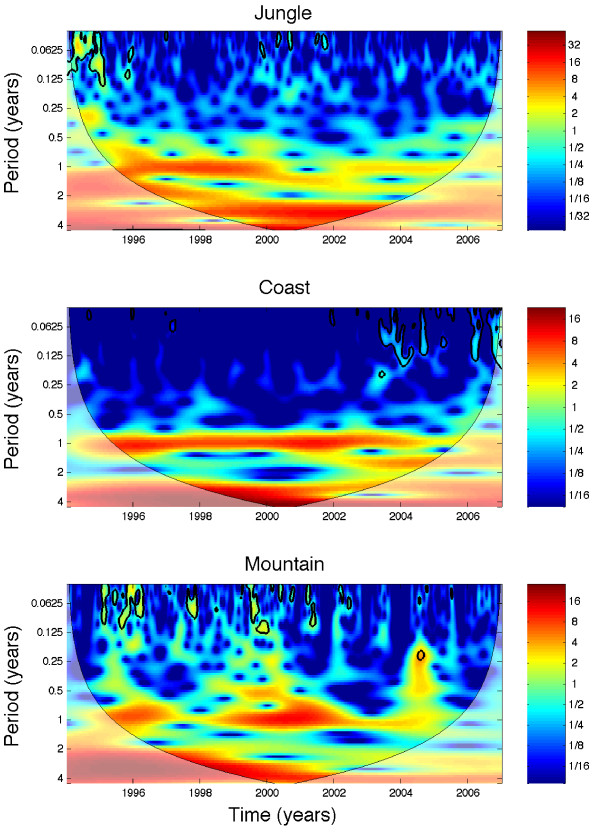
**The wavelet power spectrum of the weekly series of *P. falciparum *(1994–2006) stratified by geographic region**. In jungle regions, annual and biennial cycles dominate the beginning of the time series until about 2000 when a triennial pattern starts to show the highest power. An annual pattern in coastal regions shows almost continuously the highest power. In addition, a strong triennial pattern shows high power during 1998–2002. In mountain regions, the annual pattern shows high power during 1995–1997 and then again during 1999–2002. In addition, between 1997 and 2002 a strong triennial pattern shows high power.

Wavelet time series analysis (Figure 4) indicated that in jungle regions, annual and biennial cycles dominate the beginning of the time series of *P. falciparum *infections until about year 2000 when a stronger triennial pattern seems to emerge. In coastal regions, an annual pattern shows the highest power almost continuously. In addition, a strong triennial pattern shows high power during 1998–2002. In mountain regions, the annual pattern shows high power during 1995–1997 and again during 1999–2002. In addition, there is a strong triennial pattern between 1997 and 2002. As shown below the interpretation of these periodic patterns needs to be carried out with caution due to spatial aggregation effects.

**Figure 5:**
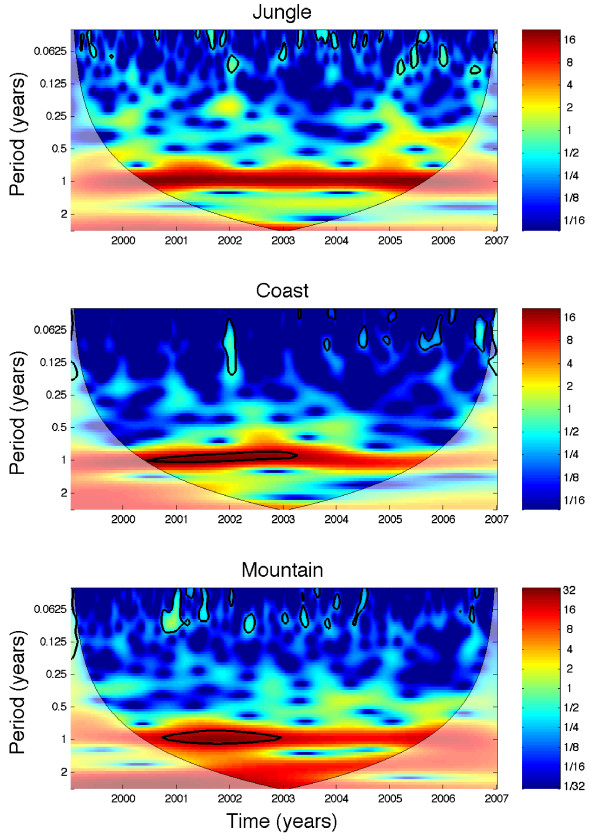
**The wavelet power spectrum of the time series of *P. vivax *(1999–2006) stratified by geographic region**. Annual cycles show a strong pattern for the three geographic regions. In addition, there is a strong biennial pattern between 2002 and 2005 in mountain regions.

For *P. vivax *(1999–2006), annual cycles show a strong pattern across geographic regions (Figure 5). Mountain regions also show a strong biennial pattern between 2002 and 2005. Moreover, the weekly incidence in the jungle was not correlated with that in the mountain range (ρ = 0.04, P = 0.38) and was weakly negatively correlated with that in the coastal region (ρ = -0.13, P = 0.008). Similarly, *P. vivax *incidence in the mountain range region was weakly negatively correlated with that in the coast (ρ = -0.13, P = 0.01) during 1999–2006. These findings are in agreement with the phase lag in the seasonality of *P. vivax *observed in the mountain regions with respect to jungle and coastal regions, as shown in Figure 3.

With regards to synchronization by geographic region, the time series of *P. falciparum *and *P. vivax *during 1999–2006 were significantly and positively correlated with each other in coastal (ρ = 0.9, P < 0.0001) and jungle regions (ρ = 0.76, P < 0.0001) and slightly correlated in mountain regions (ρ = 0.2, P < 0.0001) where sharp and brief spikes of *P. falciparum *corresponded to troughs in *P. vivax *(Figure 3).

### Temporal *P. falciparum *and *P. vivax *malaria trends and climate at the spatially refined province level

In order to better understand the factors associated with such seasonality differentials, analyses at a smaller spatial scale were carried out to look for the Provinces in each of the geographic regions where most of the malaria burden in Perú is located. The association between malaria incidence and climatological variables (mean temperature, minimum temperature, maximum temperature and precipitation) was also analysed for those 28 provinces for which weekly climatological data were available.

#### Jungle regions

The weekly malaria counts, due to *P. falciparum *and *P. vivax*, for the provinces with the highest malaria burden in jungle regions are shown in (Additional file 2 and Additional file 3), respectively. A map highlighting these provinces is given in Additional file 4. It can be seen that while *P. falciparum *is most concentrated in the northern part of the jungle, *P. vivax *extends from the northern to the southern jungle region. *P. falciparum *and *P. vivax *incidences are significantly correlated in Alto Amazonas (Spearman ρ = 0.65, P < 0.0001), Datem del Marañón (ρ = 0.71, P < 0.0001), Mariscal de Caceres (ρ = 0.80, P < 0.0001) and San Martin (ρ = 0.70, P < 0.0001). By inspection of the wavelet power spectrum at the province level, the periodicity of *P. falciparum *cases was found to be dominated by a triennial pattern to which the provinces of Alto Amazonas, Jaen, Mariscal de Caceres, and San Ignacio contribute the most (Additional file 5). Moreover, a switch from an annual to a biennial pattern in 1997 and then to a triennial pattern about 2000 can be observed in the province of Datem del Marañón. Similar changes in periodicity can be observed in the provinces of Loreto, Jaen, Utcubamba and San Ignacio. By contrast, the dynamics of *P. vivax *in jungle regions are dominated by annual cycles particularly by the province of Alto Amazonas.

In jungle regions, *P. falciparum *incidence was weakly correlated with precipitation in the province of Atalaya (Spearman ρ = 0.18, P < 0.0001). Similarly, the correlation between *P. vivax *and precipitation was weak in the province of Alto Amazonas (Spearman ρ = 0.15, P < 0.0013). Mean temperature in the province of San Martin was weakly correlated with *P. falciparum *(0.19, P < 0.0001) and *P. vivax *(0.34, P < 0.0001). Additional files 6 and 7 show the weekly time series of climatological variables and *P. falciparum *and *P. vivax *incidence in the Province of Alto Amazonas located in the jungle region.

#### Coastal regions

*Plasmodium vivax *is the dominant species in coastal areas (Additional file 4) and is highly localized in the northern coastal region. *Plasmodium falciparum *and *P. vivax *are highly synchronized in Ayabaca (Spearman ρ = 0.88, P < 0.0001), Sechura (ρ = 0.68, P < 0.0001), Paita (ρ = 0.86, P < 0.0001), Zarumilla (ρ = 0.72, P < 0.0001), Contralmirante Villar (ρ = 0.75, P < 0.0001), and Huancabamba (ρ = 0.76, P < 0.0001). The dynamics of *P. falciparum *in coastal regions are dominated by concurrent annual and triennial cycles, primarily observed in the provinces of Ayabaca, Sechura, Paita, and Zarumilla. Simpler dynamics are observed for *P. vivax *with dominant annual cycles across provinces except for a few provinces with concurrent annual and biennial cycles including Zarumilla, Lambayeque, and Ferreñafe.

Incidence in coastal areas was more closely correlated with precipitation than in jungle areas. *P. falciparum *incidence was significantly correlated with precipitation in the provinces of Chiclayo (Spearman ρ = 0.54, P < 0.0001), Ayabaca (ρ = 0.42, P < 0.0001) and Contralmirante Villar (ρ = 0.34, P < 0.0001). *P. vivax *incidence was correlated with precipitation with similar correlation coefficients (ρ = 0.32–0.48 (P < 0.0001). Furthermore, both species time series were slightly correlated with temperature variables. For example, the association with minimum temperature was highest in the province of Ascope with a correlation coefficient of 0.35 (P < 0.0001).

#### Mountain regions

The malaria burden in mountain regions has become dominated by *P. vivax *as can be seen in Figure 3. Interestingly, while *P. falciparum *is most concentrated in the northern mountain region, *P. vivax *is more concentrated in the central mountain region (Additional file 4). Moreover, *P. vivax *incidence in mountain regions has reached endemic levels with high degree of seasonality. Wavelet time series analysis on the weekly malaria time series of the 10 provinces with the highest malaria burden in mountain regions confirms the strong annual pattern observed in the analysis using aggregated time series of mountain regions, but biennial cycles, which were identified in the wavelet power spectrum using the aggregated data for mountain regions, are strong only in a few of the provinces during 2002–2004 (Additional file 8). This highlights one of the effects associated with data aggregation. This issue is described in detail in the Discussion.

There is a scarcity of climate data in the mountain regions. Moreover, the incidence of *P. falciparum *in mountain regions has been in decline in the last few years. By contrast, the dominating *P. vivax *malaria species in mountain regions was correlated with precipitation in the provinces of Cangallo (Spearman ρ = 0.25, P < 0.0001), Acomayo (ρ = 0.47, P < 0.0001) and Lauricocha (ρ = 0.46, P < 0.0001). *P. vivax *malaria incidence was moderately correlated with temperature variables (ρ = 0.29–0.47, P < 0.0001).

### Phase lag in periodic patterns

**Figure 6:**
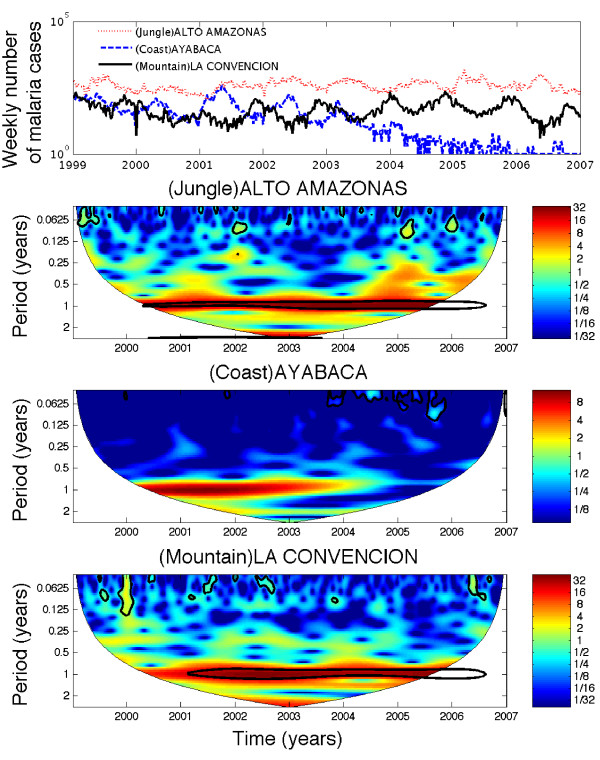
**The weekly numbers of malaria cases due to *P. vivax *in three Peruvian provinces that are representative of jungle, coast and mountain regions along with their corresponding wavelet power spectrum**. The annual pattern shows the highest power across geographic regions. While the time series of *P. vivax *in the jungle and coastal provinces follow a similar seasonality pattern, there is a phase lag in the seasonality *P. vivax *incidence in the province of La Convencion, which is representative of mountain regions.

The role played by climatological variables in contributing to the phase lag in the pattern of *P. vivax *malaria in mountain regions with respect to *P. vivax *malaria in jungle and coastal regions was assessed (Figure 3). Figure 6 shows the time series of *P. vivax *cases in three Peruvian provinces that are representative of jungle, coast and mountain regions along with their corresponding wavelet power spectrum that highlights the strong annual pattern in all three provinces.

**Figure 7:**
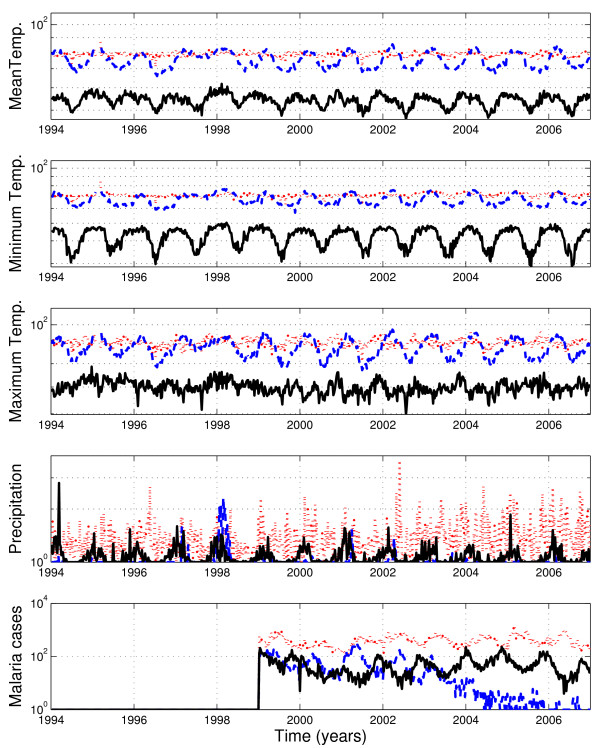
**Comparison of temperature and precipitation temporal trends and malaria cases due to *P. vivax *in three Peruvian provinces that are representative of jungle (red dotted line), coast (blue dashed line) and mountain (black solid line) regions**. The phase lag in the seasonality pattern in the Province of La Convencion, which is representative of mountain regions, with respect to the seasonality of the coastal and jungle provinces can be explained by climatological variables.

**Figure 8:**
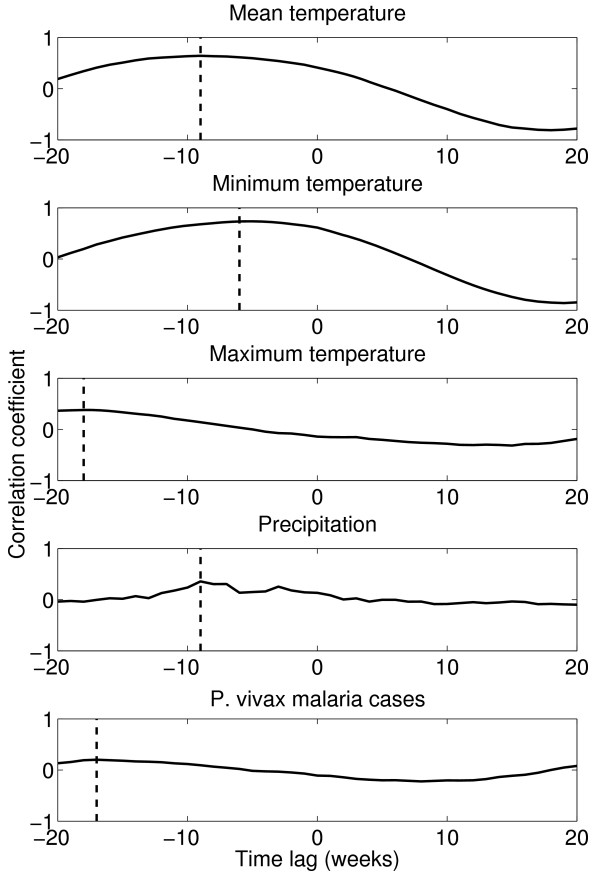
**Lagged cross-correlation plots of the climatological variables and *P. vivax *malaria incidence in the province of Ayabaca (coast) and the province of La Convencion (mountain) using the climatological data from the neighboring province of Acomayo as a proxy for the climate data for the province of La Provincia**. The dashed line indicates the temporal lag associated to the maximum correlation coefficient.

The dynamics of *P. vivax *in the province of La Convencion (mountain) follows a reciprocal seasonality pattern to that observed in Alto Amazonas (jungle) and (Ayabaca) coastal regions. A comparison of temperature and precipitation temporal trends and malaria cases due to *P. vivax *is shown in Figure 7. A lagged cross-correlation analysis indicates that climatological variables are associated with the out-phase pattern observed in mountain regions. The clearest picture was obtained from the province of Ayabaca (coast) where the climate seasonal cycle is clearly ahead of that in the mountain regions (Figure 8). Additional file 9 shows the highest correlation between precipitation and minimum temperature in the province of Acomayo (mountain) followed by Ayabaca (coast) and Alto Amazonas (jungle).

### Critical community size

While reports of *P. falciparum *were found in all the weeks during 1999–2006 in jungle regions, only 86.5% and 39.4% of the weekly time series showed *P. falciparum *reports in coastal and mountain regions, respectively. *P. vivax *is more persistent than *P. falciparum *reported in 100% of the weeks with malaria reports during 1999–2006 in jungle, mountain and coastal regions.

**Figure 9:**
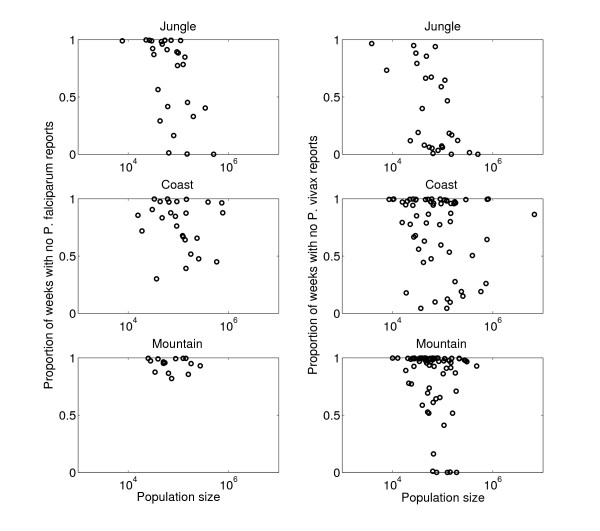
**The proportion of weeks with no malaria reports as a function of population size of the Peruvian provinces classified in coastal, mountain, and jungle areas**. The proportion of weeks with no malaria reports was negatively correlated with population size in jungle areas for both *P. falciparum *(Spearman ρ = -0.59, P = 0.001) and *P. vivax *(Spearman ρ = -0.59, P = 0.001) and in mountain regions for *P. vivax *only (Spearman ρ = -0.34, P = 0.003) during 1999–2006.

Similar persistence patterns for both *P. falciparum *and *P. vivax *malaria in jungle regions were found at the province level as measured by negative correlations between population size and the proportions of weeks in the time series with no reported malaria during the period 1999–2006 (for *P. falciparum: *Spearman ρ = -0.57, P = 0.002; for *P. vivax*: Spearman ρ = -0.48, P = 0.01, Figure 9). In addition, findings also indicated a weak but significant pattern of persistence as a function of population size for *P. vivax *in mountain regions (Spearman ρ = -0.34, P = 0.003), but this pattern was not significant for *P. falciparum*. There was no significant correlation in coastal regions between population size and the proportion of weeks with no reported malaria due to *P. falciparum *or *P. vivax*.

### Spatial heterogeneity

**Figure 10:**
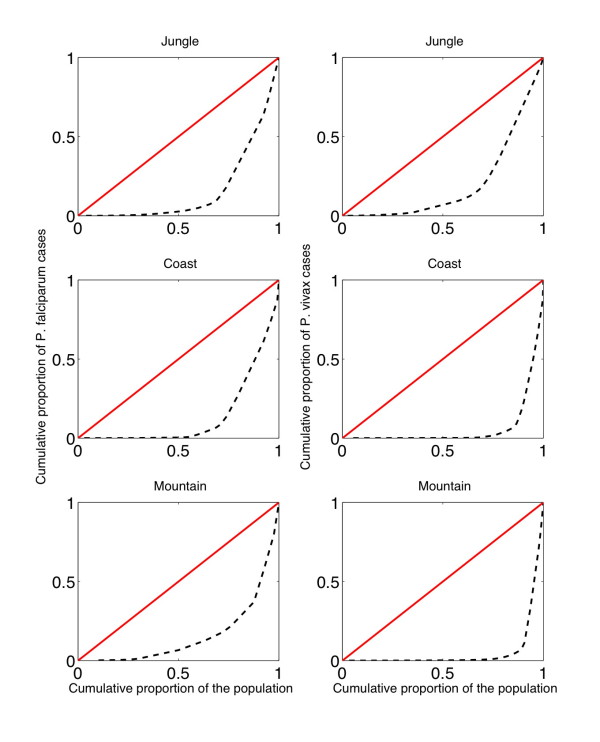
**The Lorenz curves of the distribution of the total number of malaria case notifications for *P. falciparum *and *P. vivax *as a function of population size at the province level during 1999–2006**. The red solid line (first diagonal) represents a constant distribution of malaria case notifications (no heterogeneity). During 1999–2006, spatial heterogeneity for *P. falciparum *was similar across geographic regions with the corresponding Gini indices being 0.68, 0.73, and 0.69 in jungle, coastal and mountain areas, respectively. Spatial heterogeneity for *P. vivax *in jungle regions was similar to that of *P. falciparum *during the same period (0.6) and slightly higher in mountain and coastal areas (0.89 and 0.87, respectively).

During the period 1994–1998, the spatial heterogeneity of malaria at the province level (as measured by the Gini index) due to *P. falciparum *was highest in coastal regions (0.88) followed by mountain (0.73) and jungle regions (0.57). During 1999–2006, spatial heterogeneity for *P. falciparum *was similar across geographic regions with the corresponding Gini indices being 0.68, 0.73, and 0.69 in jungle, coastal and mountain areas, respectively. That is, the Gini index "converged" from 0.88/0.73/0.57 to 0.73/0.69/0.68 in coastal, mountain and jungle areas. Spatial heterogeneity for *P. vivax *in jungle regions was similar to that of *P. falciparum *during the same period (0.6) and slightly higher in mountain and coastal areas (0.89 and 0.87, respectively). Figure 10 shows the corresponding Lorenz curves by geographic region for the common period 1999–2006.

## Discussion

Although a few studies have explored spatio-temporal patterns of malaria in regions such as Thailand [27], and global maps of malaria risk have started to be generated [28] to the best of knowledge this is the first study to assess the spatial and temporal dynamics of malaria using high-resolution spatio-temporal data of *P. falciparum *and *P. vivax *malaria infections in South America.

Despite the declining trend of *P. falciparum*, both *P. falciparum *and *P. vivax *weekly counts were found to be strongly correlated in jungle and coastal areas and weakly correlated in mountain regions where *P. falciparum *infections have become rare in the last few years. However, in those regions sharp and brief spikes of *P. falciparum *infections concur with troughs of *P. vivax *malaria. In contrast, the time series of *P. falciparum *and *P. vivax *malaria at the national level were only moderately correlated. This highlights the importance of high-resolution spatio-temporal data to detect heterogeneity in malaria incidence and periodicity patterns.

While analysis of temporal malaria trends of *P. vivax *malaria at the aggregated national level are quite consistent with the corresponding analyses by geographic region or province level (strong annual pattern followed by biennial cycles), results indicate more irregular periodicity patterns for *P. falciparum *where biennial and triennial cycles have emerged in the more recent years of the study period, probably due to its significant decline, particularly in mountain and coastal regions. This highlights the role of spatial scales in the periodicity of malaria incidence [29]. Two types of problems derived from using aggregated data were identified. First, dominant periodic patterns could differ across spatial units (e.g., provinces). For example, in this study, wavelet time series analysis identified a single dominant triennial pattern due to *P. falciparum *in the jungle provinces of Alto Amazonas, Jaen, Mariscal de Caceres, and San Ignacio, but this is not the characteristic pattern observed from the jungle-region-level wavelet time series analysis. Thus, apparent changes could be detected in periodicity over time in jungle regions while ignoring true local differences in periodicity at the province level (Figure 4). These differences in periodicity at different spatial scales could be associated with variations in the environmental landscape such as floods in the highly heterogeneous network of rivers and lakes [3,30,31]. Furthermore, the aggregated data for *P. vivax *in coastal regions indicate a continuously strong annual pattern, whereas wavelet spectral analysis at the province level reveals a few provinces with concurrent annual and biennial cycles including Zarumilla, Lambayeque, and Ferreñafe. Another complication in understanding of the spatio-temporal processes involved in malaria transmission arises when incidence in two or more localities are phase lagged with respect to each other. In Perú, the time series of malaria due to *P. vivax *in mountain regions were found to be significantly phase lagged (about 180 degrees) with respect to coast and jungle areas. Importantly, this pattern could not have been deciphered from the time series aggregated at the national level alone. In regards to phase lags in the incidence of malaria, a similar pattern has been reported for the archipelago of Vanuatu where *P. falciparum *malaria was found to be predominant in the long wet season and *P. vivax *malaria was predominant in the dry season [32,33]. Although the best climatological data at the province level available for Perú were used, these results need to be interpreted with caution. Climatological data from meteorological stations may not be necessarily representative for the larger provinces and the station coverage in the mountain regions was limited. The use of interpolated data with better meteorological station coverage at smaller spatial scales (e.g., district level) is recommended.

Understanding variability in the temporal dynamic of malaria epidemics has public health implications. For example, a number of studies have explored the roles of exogenous (e.g., climate) and endogenous (e.g., intrinsic to the transmission dynamics) factors in this variability using monthly data from East Africa [34-36]. The current understanding is that rainfall and temperature play an important role in cycles of low periodicity, while endogenous dynamics of malaria may explain cycles of longer periodicity [23]. The findings of this study indicate moderate levels of correlation between malaria incidence and precipitation in coastal areas; however, this correlation was weak in jungle areas. The lower correlation in jungle areas could be explained by significant heterogeneity in the dynamics of breeding sites due to the presence of flooding of the surroundings of the highly heterogeneous river networks and lakes [3,30,31] and population mobility patterns. For example, anecdotal evidence by one of the team members (CM) suggests that outbreaks of malaria infections in northern jungle regions of Perú are associated with movement patterns of timber merchants who enter the jungle at low river levels and exit when river levels rise. In fact, a study has found that the daily Nanay river level in Iquitos, Perú to explain 28% of the variability in total malaria risk [30,3]. Similarly, other activities associated with malaria infection risk are the gold mining industry in the Southern jungle areas confined mainly to the beaches of the Inambari and Madre de Dios rivers, and rice production in the northern coastal areas of Perú due to inappropriate cleaning of the irrigation systems [37].

The decline in the incidence of *P. falciparum *is encouraging, but it needs to be interpreted with caution as it could be associated with multiple factors including methodological (e.g., associated with microscopy-based identification of malaria species [38-40]), treatment interventions [41,42] and cross-protective immunity [43]. Traditional microscopy-based detection techniques have significant limitations by failing to detect the presence of low-level and mixed infections [38,39,44]. In fact, literature reviews have concluded that in general fewer mixed species *P. falciparum/P. vivax *infections are observed than would be expected from the prevalence of constituent malaria species, which is in agreement with this study [45,46,43]. Longitudinal field studies [47-50] and clinical studies (e.g., [50]) suggest that in mixed-species malaria infections in humans one of the constituent *Plasmodium *species tends to dominate. Nevertheless, the consistent and gradual decline of the incidence of *P. falciparum *across geographic regions puts more weight into the role of their biological differences leading to different treatment interventions and cross-protective immunity.

Unlike *P. falciparum *infections, a significant fraction of *P. vivax *cases relapse following treatment due to the difficulty in completely eradicating dormant liver stages of the parasite (the hypnozoites), those stages are targeted by Primaquine. Hence, *P. vivax *incidence could be affected by relapses of previously infected individuals [51]. While the *P. vivax *treatment guidelines have not had significant changes for almost 50 years (with first-line therapies, Chloroquine+Primaquine, unchanged for 50 years) [52], there have been changes in malaria treatment policies for *P. falciparum *driven primarily by the appearance of drug resistance [53], such changes may have affected the observed incidence of *P. falciparum*. Indeed, the spike of *falciparum *malaria in the 90's has been explained in part by the use of ineffective drugs.

In addition, the role of cross-protective immunity whereby prior exposure to *P. vivax *infection ameliorates the course of a subsequent *P. falciparum *infection could also contribute to the substantial reduction of *P. falciparum *infections [31,54]. Hence, further work that includes longitudinal studies that make use of accurate detection methods [55,56] across geographic regions of Perú are needed to quantify the impact of pharmaceutical interventions and cross-protection immunity against malaria. Further insights into the dynamics and impact of interventions can be gained through the use of mathematical models [57].

Important differences in the dynamic of *P. falciparum *and *P. vivax *were found in Perú beyond the fact that *P. vivax *is more widespread in Perú than *P. falciparum*. Of note, community size was also found to be significantly associated with malaria persistence due to both malaria species in jungle regions but not in coast and mountain regions, which is in agreement with dengue persistence in Perú [58]. Hence, this highlights the need to evaluate the impact of malaria control programmes in jungle areas and the potential benefits from the use of bed nets as a means of reducing the "effective community size" in areas where malaria persistence is highest. It is worth pointing out that the authors have made use of the best, most complete data available, and while of course it is not ideal, it is reassuring that the patterns of persistence as a function of community size are quite clear in jungle areas which are the most remote and difficult to access in Perú.

Findings indicated significant spatial heterogeneity due to *P. falciparum *(1999–2006) with similar levels across geographic regions (Gini ~ 0.7), which could suggest important differences in levels of control of *P. falciparum *in all geographic regions. Similarly, spatial heterogeneity associated to *P. vivax *was higher in coastal (0.87) and mountain (0.89) regions while heterogeneity in jungle regions (0.6) was similar to that of *P. falciparum *infections, indicating less variability in the activity of *P. vivax *in jungle than in coastal or mountain regions. For comparison, spatial heterogeneity of dengue fever (1994–2006) in Perú has been reported to be higher in coastal areas (Gini ~ 0.59), followed by mountain (0.36) and jungle areas (0.27) [58].

## Conclusion

Overall, this study highlights the importance of highly refined spatial and temporal data of malaria incidence together with demographic and geographic information in improving the understanding of malaria persistence due to multiple malaria species in human populations, the impact of pharmaceutical interventions, detection of heterogeneity, and generation of hypotheses.

## Competing interests

The authors declare that they have no competing interests.

## Authors' contributions

GC conceived the study, analysed the data and wrote the first draft of the manuscript. CM, AE and FEM participated in the interpretation of results and in the writing and editing of the manuscript.

## Supplementary Material

Additional file 1 **Distribution of population density across coastal, mountain and jungle areas in Perú**. Boxplot of the distribution of population density in coastal, mountain, and jungle areas. The population density ranged from a median of 22.3 people/km^2 ^in the mountain range, 12.38 in the jungle areas, and 172 in the coastal areas. Click here for file

Additional file 2 **The weekly malaria counts for the provinces with the highest *P. falciparum *malaria burden in jungle regions**. The weekly malaria counts for the provinces with the highest *P. vivax *malaria burden in jungle regions. *P. falciparum *and *P. vivax *incidence is significantly correlated in Alto Amazonas (Spearman rho = 0.65, P < 0.0001), Datem del Marañón (rho = 0.71, P < 0.0001), Mariscal de Caceres (rho = 0.80, P < 0.0001) and San Martin (rho = 0.70, P < 0.0001). Case notification of *P. vivax *did not start until 1999. Click here for file

Additional file 3 **The weekly malaria counts for the provinces with the highest *P. vivax *malaria burden in jungle regions**. The weekly malaria counts for the provinces with the highest *P. vivax *malaria burden in jungle regions. *P. falciparum *and *P. vivax *incidence is significantly correlated in Alto Amazonas (Spearman rho = 0.65, P < 0.0001), Datem del Marañón (rho = 0.71, P < 0.0001), Mariscal de Caceres (rho = 0.80, P < 0.0001) and San Martin (rho = 0.70, P < 0.0001). Case notification of *P. vivax *did not start until 1999. Click here for file

Additional file 4 **Distribution of *P. falciparum *and *P. vivax *across geographic regions in Perú**. The ten provinces with the highest malaria burden due to *P. falciparum *and *P. vivax *are highlighted with red squares (jungle), triangles (mountain) and circles (coast). Black dots indicate the centroides (latitude, longitude coordinates) of each of the 195 provinces comprising jungle, coastal and mountain regions. Click here for file

Additional file 5 **Wavelet power spectrum of *P. falciparum *time series in jungle regions**. The wavelet power spectrum of the time series of *P. falciparum *(1994–2006) for the provinces with the highest *P. falciparum *malaria burden in jungle regions. Click here for file

Additional file 6 **Weekly time series of *P. falciparum *malaria and climatological variables in the province of Alto Amazonas**. The weekly time series of four climatological variables: mean temperature (°F), minimum temperature (°F), maximum temperature (°F), precipitation (in) and *P. falciparum *malaria incidence in the province of Alto Amazonas, which is located in the jungle, during 1994–2006. Click here for file

Additional file 7 **Weekly time series of *P. vivax *malaria and climatological variables in the province of Alto Amazonas**. The weekly time series of four climatological variables: mean temperature (°F), minimum temperature (°F), maximum temperature (°F), precipitation (in) and *P. vivax *malaria incidence in the province of Alto Amazonas, which is located in the jungle, during 1999–2006. Click here for file

Additional file 8 **Wavelet power spectrum of *P. falciparum *time series in mountain regions**. The wavelet power spectrum of the time series of *P. vivax *(1999–2006) for the provinces with the highest *P. vivax *malaria burden in mountain regions. Whereas annual cycles are the dominant pattern, strong biennial cycles can only be observed in a few of the provinces in mountain regions. Click here for file

Additional file 9 **Correlation between precipitation and minimum temperature in three provinces representative of jungle, coastal and mountain regions**. The correlation between precipitation and minimum temperature in three provinces that representative of jungle (Alto Amazonas), coastal (Ayabaca) and mountain (Acomayo) regions in Perú. The blue dashed line is a spline curve to highlight a significant correlation trend. Click here for file
