# Supplementary figures and images for: The spatial and temporal patterns of falciparum and vivax malaria in Perú: 1994–2006
Source: Malar J. 2009 Jun 27;8:142. doi: 10.1186/1475-2875-8-142 (PMC2714521; doi:10.1186/1475-2875-8-142)

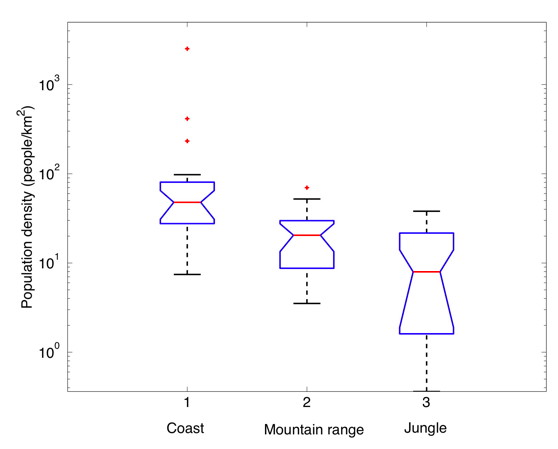

Supplement: Additional file 1 — Distribution of population density across coastal, mountain and jungle areas in Perú. Boxplot of the distribution of population density in coastal, mountain, and jungle areas. The population density ranged from a median of 22.3 people/km2 in the mountain range, 12.38 in the jungle areas, and 172 in the coastal areas. [file 1475-2875-8-142-S1.jpeg]

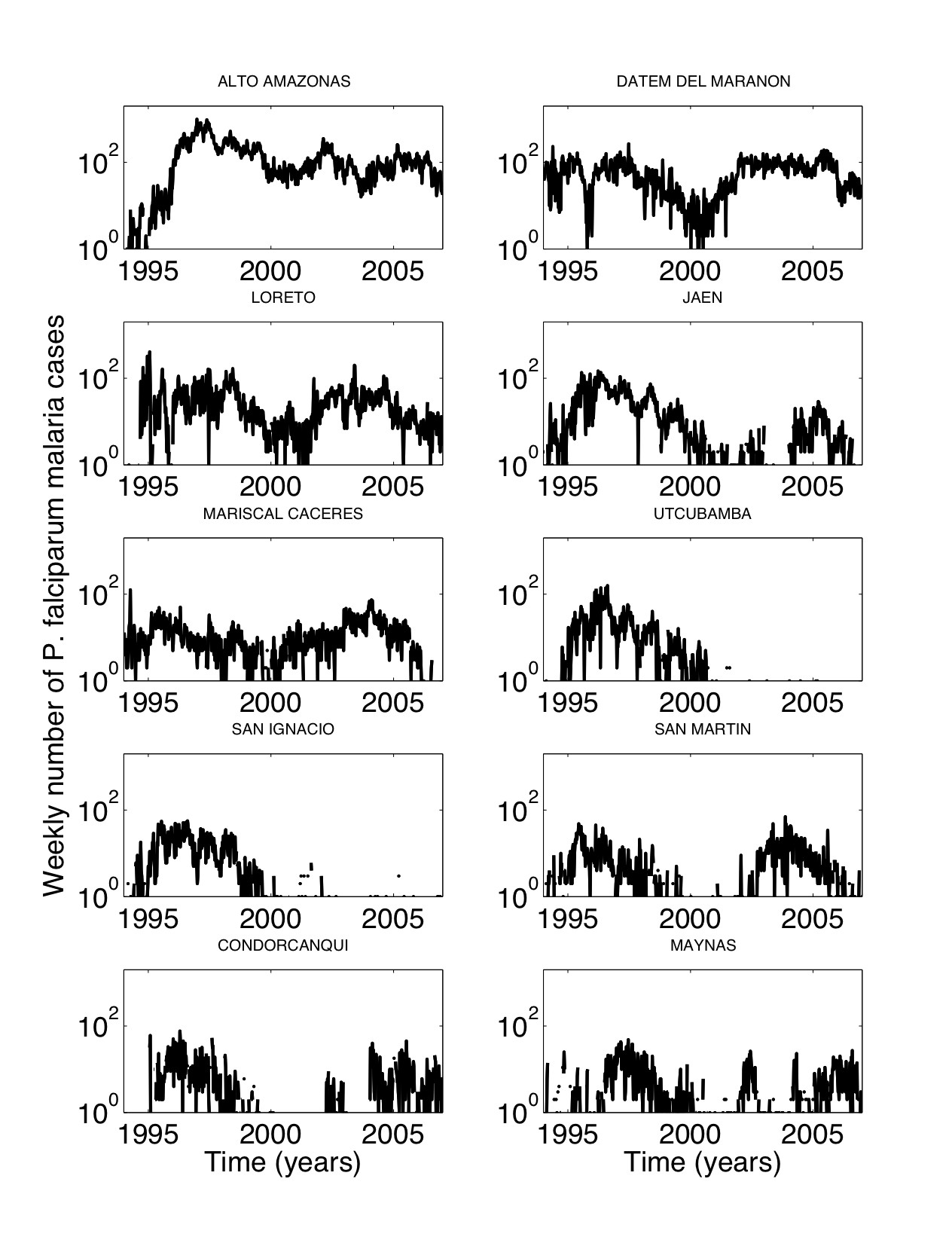

Supplement: Additional file 2 — The weekly malaria counts for the provinces with the highest P. falciparum malaria burden in jungle regions. The weekly malaria counts for the provinces with the highest P. vivax malaria burden in jungle regions. P. falciparum and P. vivax incidence is significantly correlated in Alto Amazonas (Spearman rho = 0.65, P < 0.0001), Datem del Marañón (rho = 0.71, P < 0.0001), Mariscal de Caceres (rho = 0.80, P < 0.0001) and San Martin (rho = 0.70, P < 0.0001). Case notification of P. vivax did not start until 1999. [file 1475-2875-8-142-S2.jpeg]

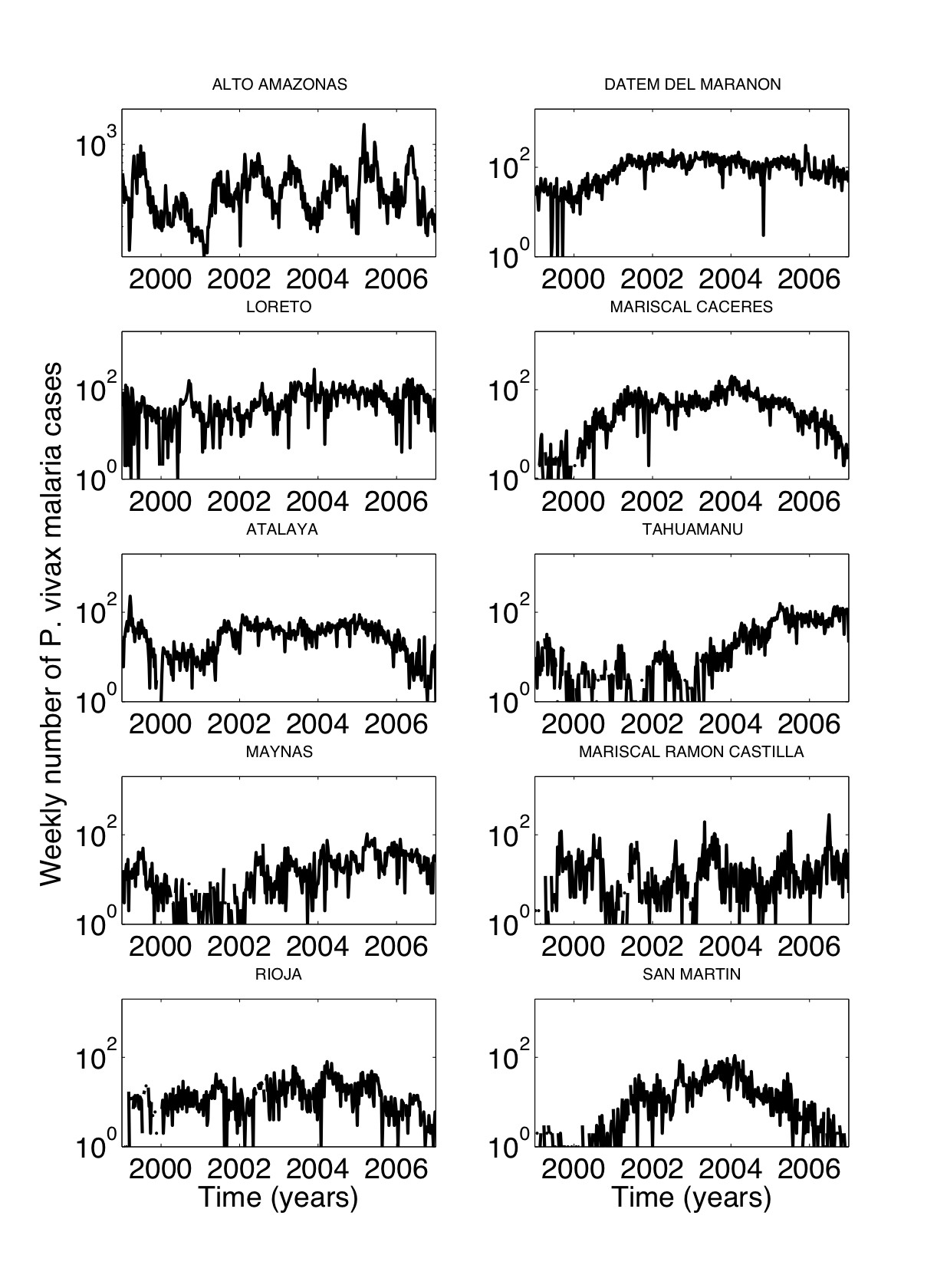

Supplement: Additional file 3 — The weekly malaria counts for the provinces with the highest P. vivax malaria burden in jungle regions. The weekly malaria counts for the provinces with the highest P. vivax malaria burden in jungle regions. P. falciparum and P. vivax incidence is significantly correlated in Alto Amazonas (Spearman rho = 0.65, P < 0.0001), Datem del Marañón (rho = 0.71, P < 0.0001), Mariscal de Caceres (rho = 0.80, P < 0.0001) and San Martin (rho = 0.70, P < 0.0001). Case notification of P. vivax did not start until 1999. [file 1475-2875-8-142-S3.jpeg]

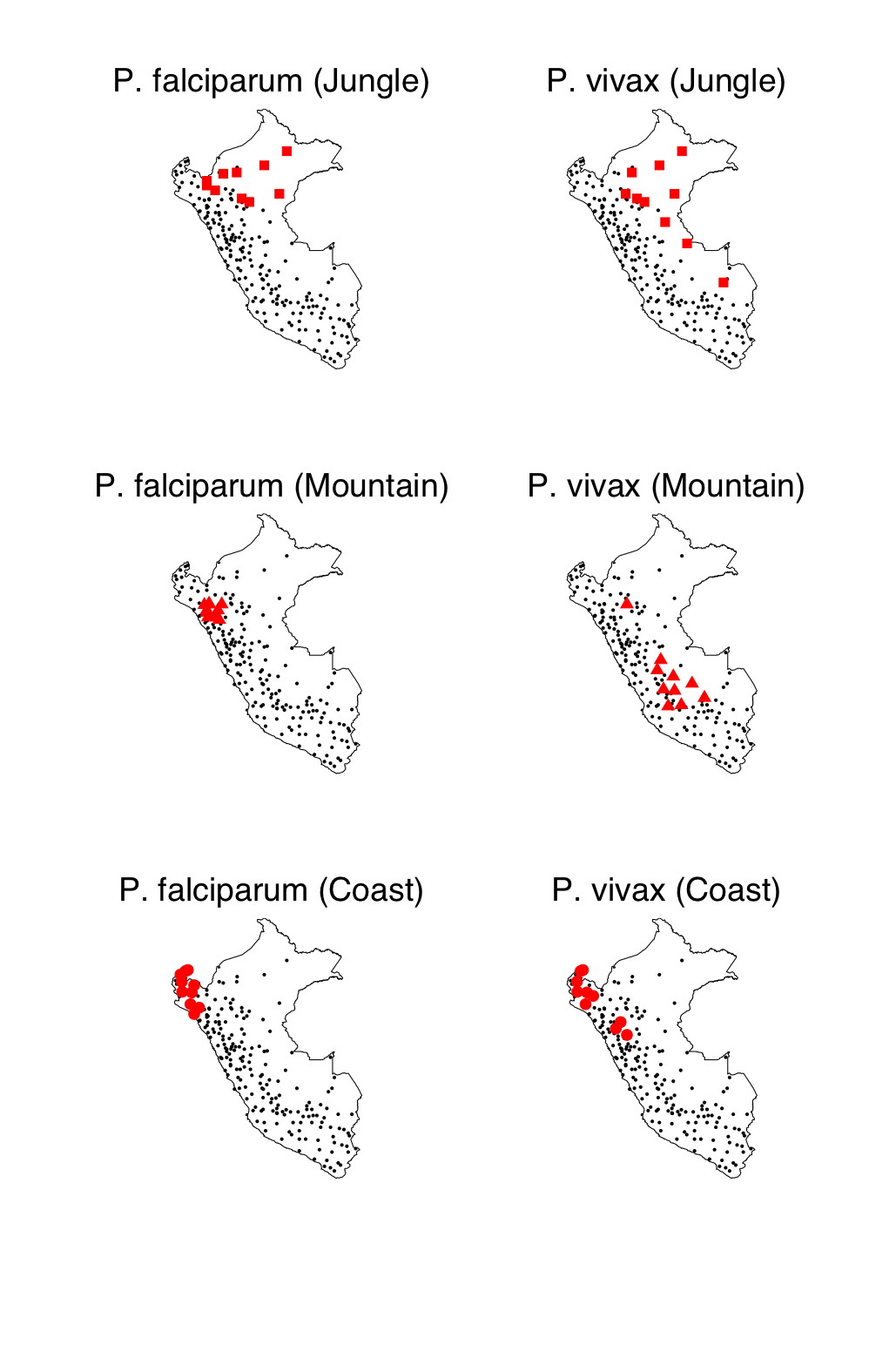

Supplement: Additional file 4 — Distribution of P. falciparum and P. vivax across geographic regions in Perú. The ten provinces with the highest malaria burden due to P. falciparum and P. vivax are highlighted with red squares (jungle), triangles (mountain) and circles (coast). Black dots indicate the centroides (latitude, longitude coordinates) of each of the 195 provinces comprising jungle, coastal and mountain regions. [file 1475-2875-8-142-S4.jpeg]

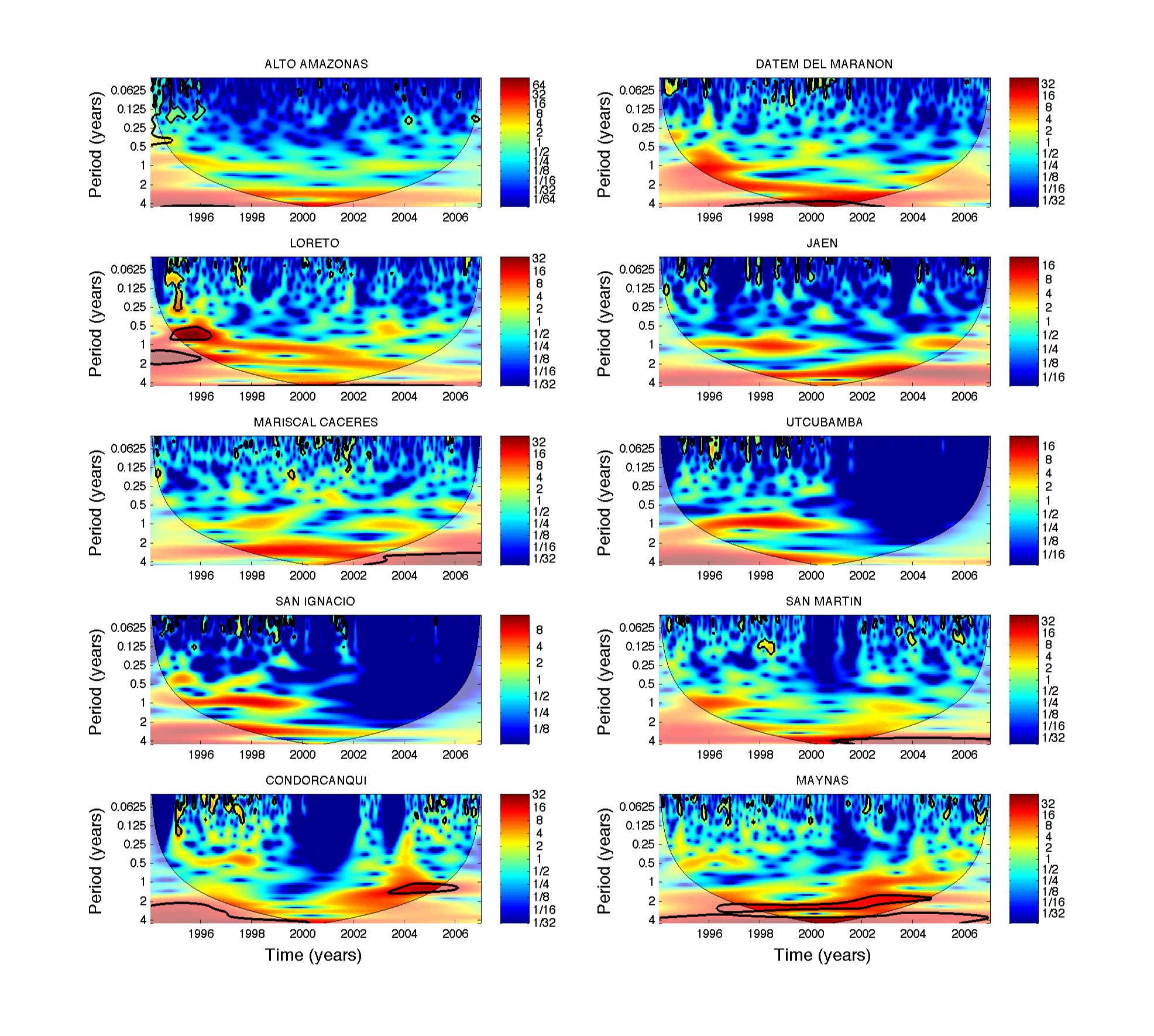

Supplement: Additional file 5 — Wavelet power spectrum of P. falciparum time series in jungle regions. The wavelet power spectrum of the time series of P. falciparum (1994–2006) for the provinces with the highest P. falciparum malaria burden in jungle regions. [file 1475-2875-8-142-S5.jpeg]

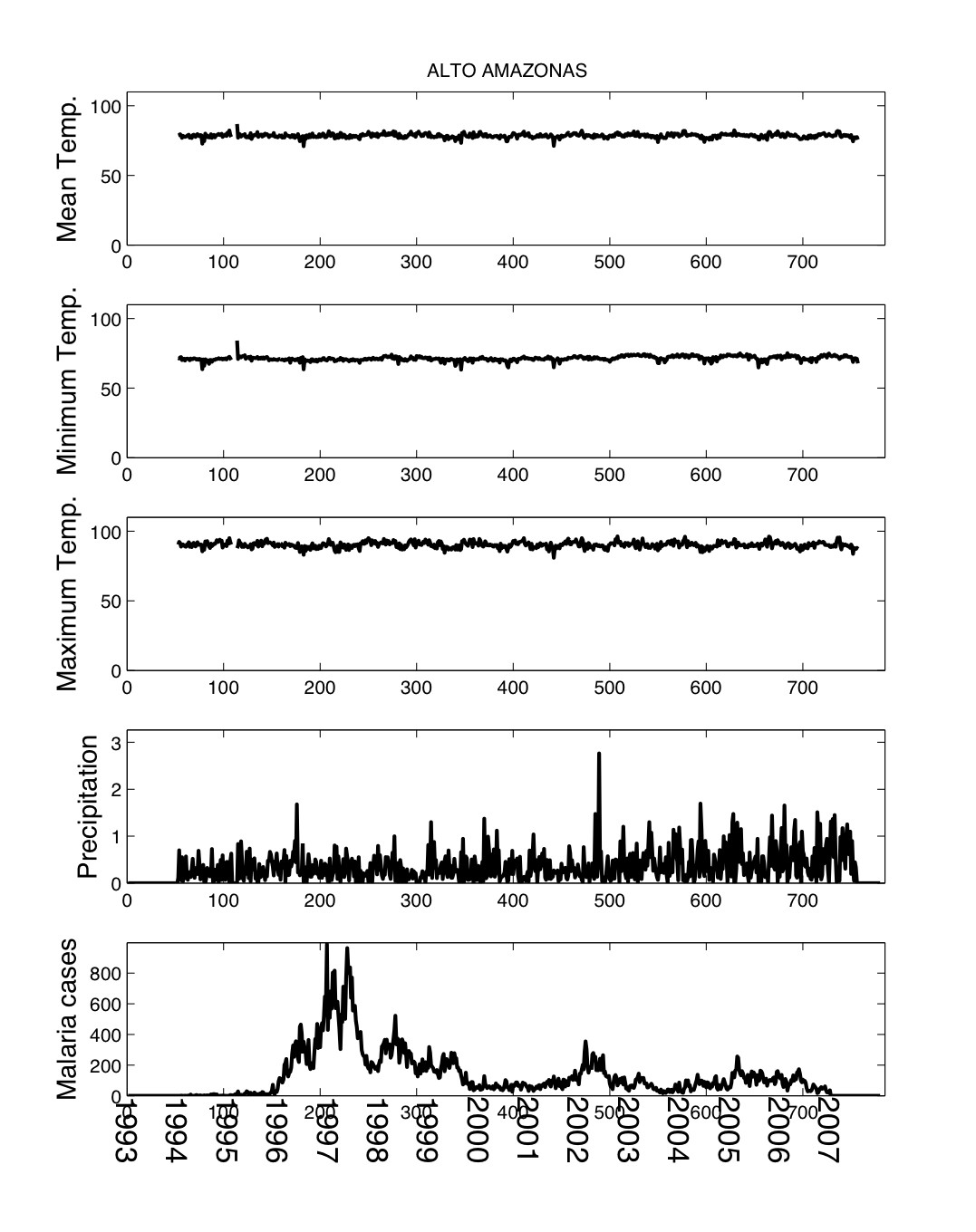

Supplement: Additional file 6 — Weekly time series of P. falciparum malaria and climatological variables in the province of Alto Amazonas. The weekly time series of four climatological variables: mean temperature (°F), minimum temperature (°F), maximum temperature (°F), precipitation (in) and P. falciparum malaria incidence in the province of Alto Amazonas, which is located in the jungle, during 1994–2006. [file 1475-2875-8-142-S6.jpeg]

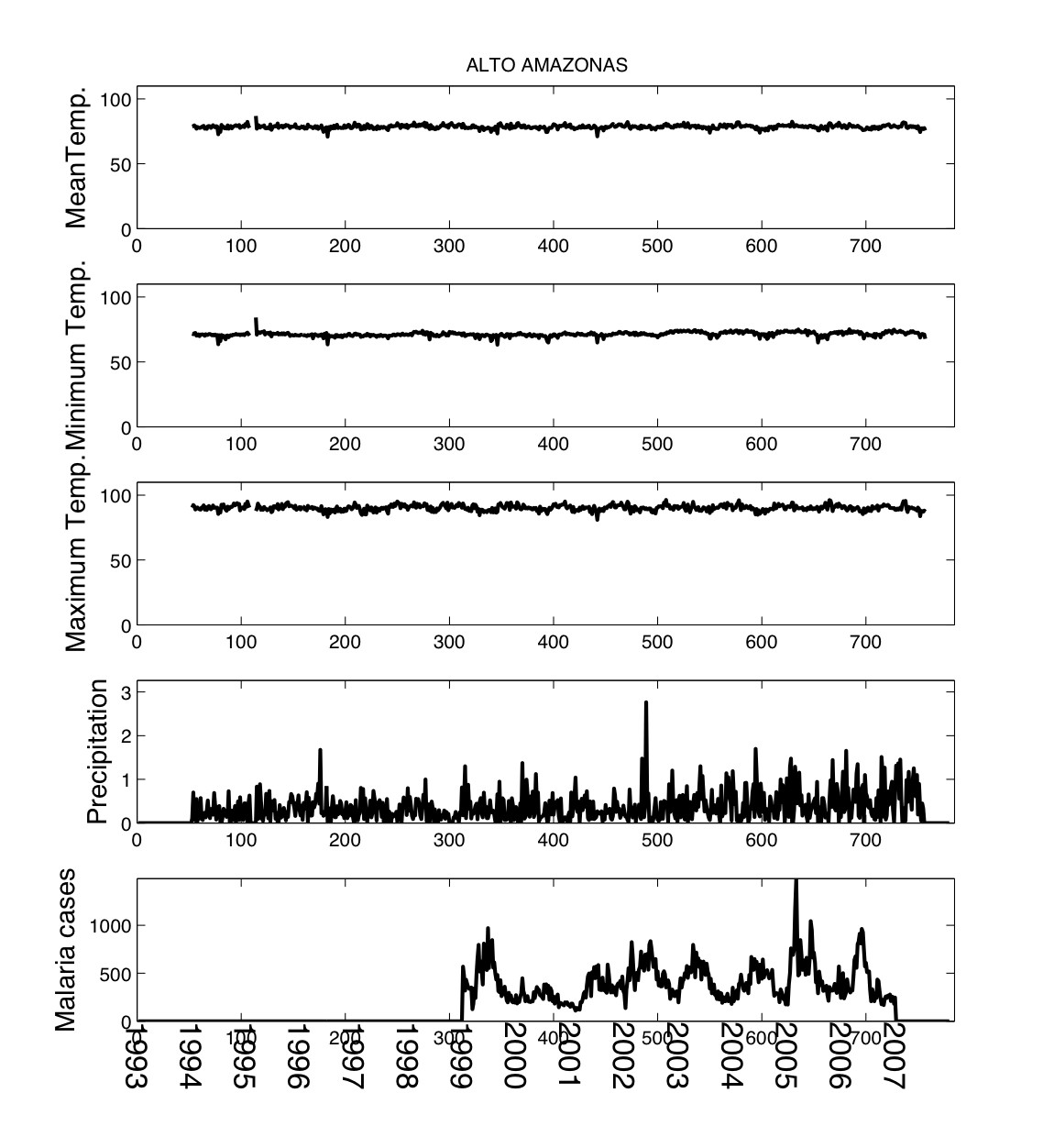

Supplement: Additional file 7 — Weekly time series of P. vivax malaria and climatological variables in the province of Alto Amazonas. The weekly time series of four climatological variables: mean temperature (°F), minimum temperature (°F), maximum temperature (°F), precipitation (in) and P. vivax malaria incidence in the province of Alto Amazonas, which is located in the jungle, during 1999–2006. [file 1475-2875-8-142-S7.jpeg]

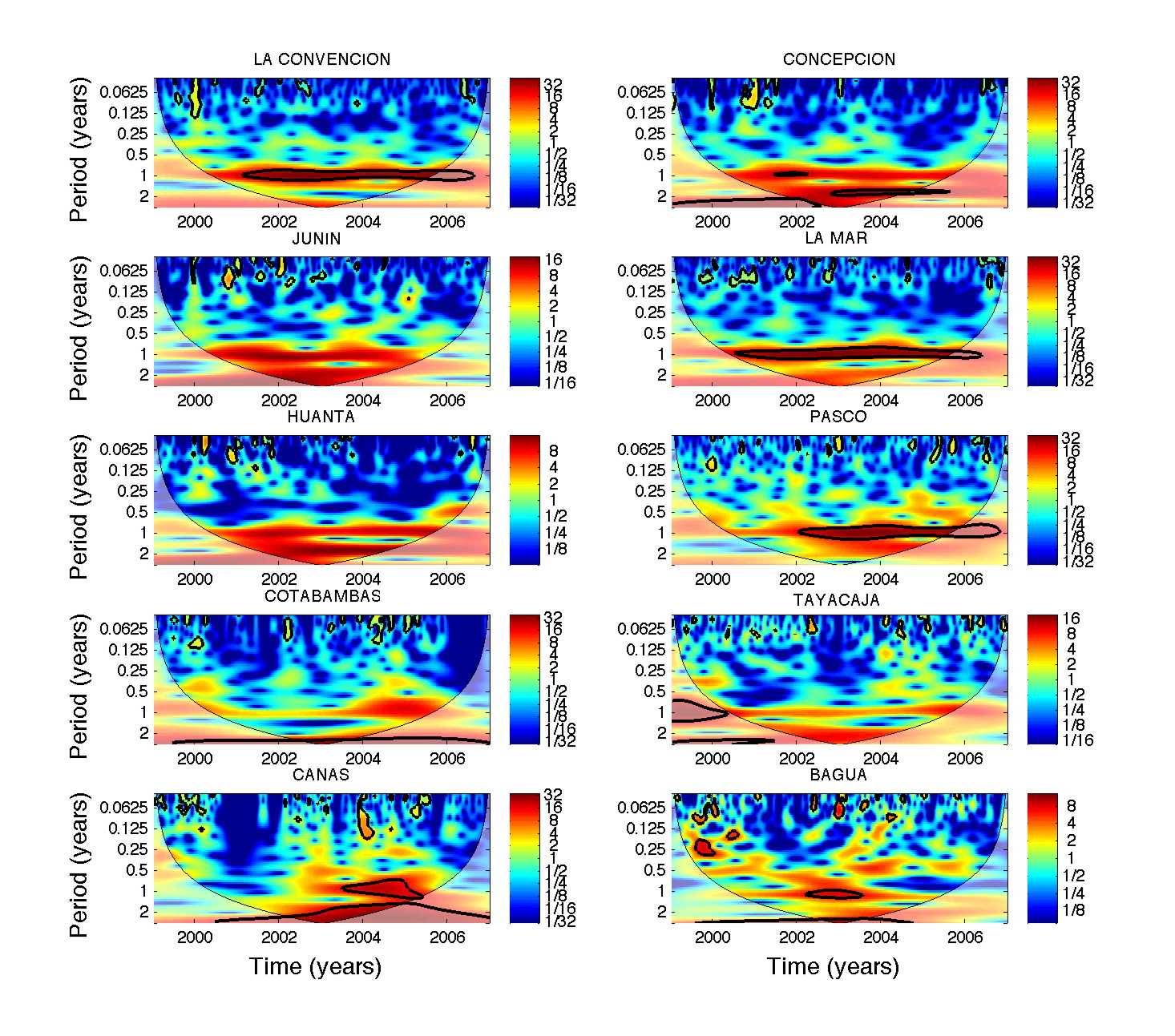

Supplement: Additional file 8 — Wavelet power spectrum of P. falciparum time series in mountain regions. The wavelet power spectrum of the time series of P. vivax (1999–2006) for the provinces with the highest P. vivax malaria burden in mountain regions. Whereas annual cycles are the dominant pattern, strong biennial cycles can only be observed in a few of the provinces in mountain regions. [file 1475-2875-8-142-S8.jpeg]

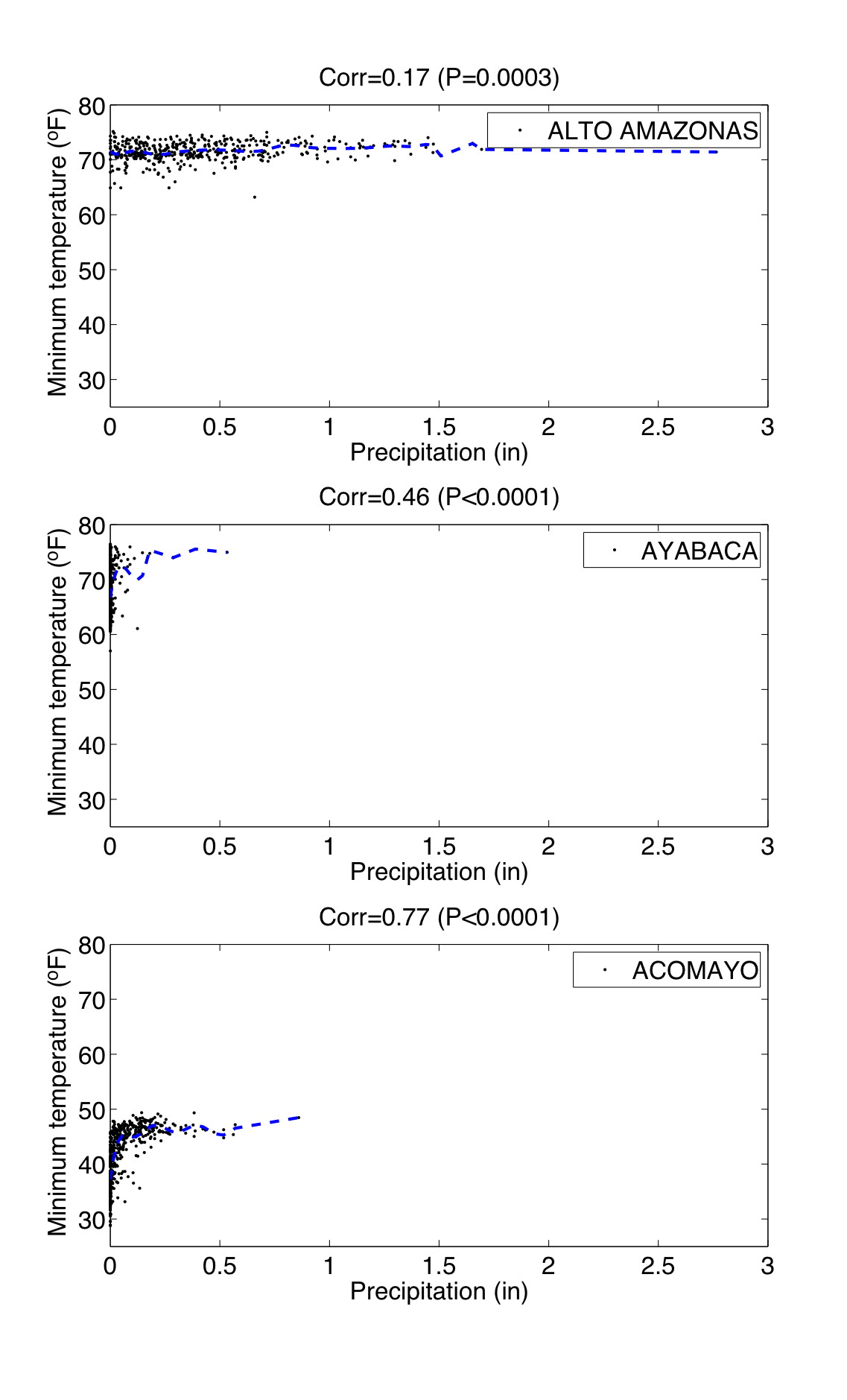

Supplement: Additional file 9 — Correlation between precipitation and minimum temperature in three provinces representative of jungle, coastal and mountain regions. The correlation between precipitation and minimum temperature in three provinces that representative of jungle (Alto Amazonas), coastal (Ayabaca) and mountain (Acomayo) regions in Perú. The blue dashed line is a spline curve to highlight a significant correlation trend. [file 1475-2875-8-142-S9.jpeg]
